# A case of late-onset multiple sclerosis mimicking glioblastoma and displaying intraoperative 5-aminolevulinic acid fluorescence

**DOI:** 10.1007/s00701-012-1319-z

**Published:** 2012-03-09

**Authors:** U. Nestler, A. Warter, P. Cabre, N. Manzo

**Affiliations:** 1 Department of Neurosurgery, Service 6B, Centre Hospitalier Universitaire, BP 632, 97200 Fort-de-France, France; 2 Department of Pathology, Centre Hospitalier Universitaire de Fort de France, Fort-de-France, France; 3 Department of Neurology, Centre Hospitalier Universitaire de Fort de France, Fort-de-France, France

Dear Editor,

The resemblance of multiple sclerosis lesions to brain tumours is encountered in about 1% of cases and differentiation between these diagnoses remains difficult [2, 10]. In tumours, administration of 5-aminolevulinic acid (5-ala) leads to accumulation of porphyrinogens, which can be detected intraoperatively by fluorescence [7]. In contrast to this, little is known about the staining properties of non-neoplastic intracranial lesions [5]. Multiple sclerosis is an autoimmune, chronic inflammatory disease and its first occurrence beyond the age of 50 is rare [4].

We describe the case of a 57-year-old banana plantation worker with right-sided weakness, trouble in finding words and an organic psychosyndrome. Even though receiving corticosteroids, his symptoms aggravated.

**Fig. 1:**
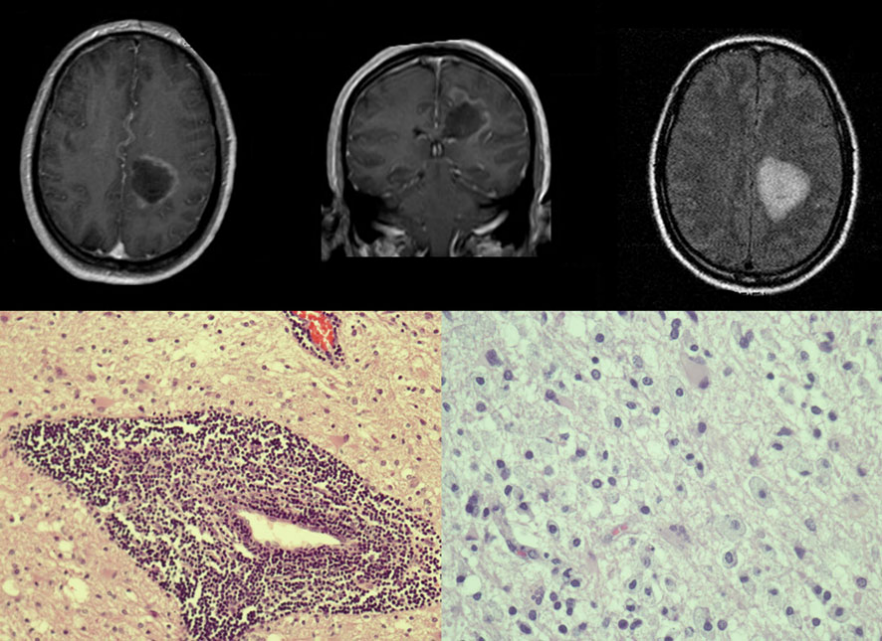
*Upper row*: preoperative MRI with axial T1 gadolinium-enhanced, coronal T1 gadolinium-enhanced and axial FLAIR images. *Lower row left*: lymphocytic cuffs in the spaces of Virchow-Robin (haematoxylin and eosin stain [H&E], original magnification ×20). *Lower row right*: reactive astrocytes, in the lower middle a multinucleated cell, and infiltration of macrophages (H&E, original magnification ×40)

Magnetic resonance imaging (MRI) disclosed a single, left parietal periventricular lesion with a diameter of 34 mm and displaying rim-like contrast enhancement without significant mass effect (Fig. 1).

During resection, clear 5-ala fluorescence was observed, useful for defining resection margins. The ventricular wall was opened and no fluorescence was found intraventricularly nor near to the choroid plexus.

Intraoperative histology was compatible with astrocytoma. Final histological examination disclosed lymphocytic cuffs in the Virchow-Robin spaces, reactive astrocytes, partly with multiple nuclei, and abundant infiltrations of macrophages (Fig. 1). A second opinion from Mme. Prof. Daumas Duport confirmed the diagnosis of multiple sclerosis. Lumbar puncture then revealed local synthesis of IgG and IgA, an oligoclonal IgG aspect on isofocalisation and signs of altered blood-brain barrier.

Five months after surgery, the patient suffered from slightly spastic hemiparesis, with ability to walk a few steps and to grasp and hold objects with the right hand. MRI control did not detect any sclerotic plaques nor gadolinium-enhancing lesions.

Although diagnostic problems between multiple sclerosis and glioma have been described before [2], this case remains remarkable in several aspects. The late onset of multiple sclerosis often follows a primary progressive course [3]. Similar to our patient, most cases become symptomatic with motor deficits, whereas rim-like focal gadolinium enhancement on MRI with absence of multifocal lesions is rare [4]. Generally, unlike this case, the symptoms respond well to corticosteroid treatment.

Differentiation between multiple sclerosis and acute demyelinating encephalomyelitis (ADEM) at first onset in the adult age is under debate [8]. In our case, the histological pattern, the absence of recent infection and the triad of clinical symptoms, oligoclonal bands in cerebrospinal fluid (CSF) and white matter lesion, were in favour of multiple sclerosis [3]. In another non-neoplastic 5-ala-fluorescence-positive case, Behcet’s disease has been discussed [5].

Few reports deal with the relationship between activated lymphocytes and the porphobilinogen pathway [6, 9]. Concerning malignant lymphoma, theoretical considerations of deficits in ferrochelatase enzymatic activity, leading to accumulation of protoporphyrin IX and fluorescence, have been confirmed in cell culture and during neurosurgical interventions [1, 6].

In our case, the occurrence of intraoperative fluorescence can be explained by functional inhibition of ferrochelatase activity in activated lymphocytes [6]. The activated lymphatic cells increase their metabolism and their proliferative potential, for both of which iron is required. This leads to an iron shortage in the ferrochelatase step of haeme synthesis, thus resulting in upstream accumulation of 5-ala-induced protoporphyrin IX and giving rise to fluorescence.

5-Ala-induced fluorescence is not restricted to malignant tumour cells. Cells with an inhibition of the ferrochelatase pathway, such as activated lymphocytes, are prone to accumulate protoporphyrin IX. Solitary multiple sclerosis plaques thus remain a rare, but important differential diagnosis of malignant glioma.
